# A novel, multiplex, real-time PCR–based approach for the detection of the commonly occurring pathogenic fungi and bacteria

**DOI:** 10.1186/1471-2180-13-300

**Published:** 2013-12-23

**Authors:** Ádám Horváth, Zoltán Pető, Edit Urbán, Csaba Vágvölgyi, Ferenc Somogyvári

**Affiliations:** 1 Department of Medical Microbiology and Immunobiology, Faculty of Medicine, University of Szeged, Dóm tér 10, Szeged H-6720, Hungary; 2 Department of Anaesthesiology and Intensive Therapy, Faculty of Medicine, University of Szeged, Semmelweis u. 6, Szeged, Hungary; 3 Institute of Clinical Microbiology, Faculty of Medicine, University of Szeged, Semmelweis u. 6, Szeged, Hungary; 4 Department of Microbiology, Faculty of Science and Informatics, University of Szeged, Közép fasor 52, Szeged, Hungary

**Keywords:** Clinically relevant bacteria, *Candida*, Melting point analysis, Fluorescence resonance energy transfer, Bloodstream infections

## Abstract

**Background:**

Polymerase chain reaction (PCR)-based techniques are widely used to identify fungal and bacterial infections. There have been numerous reports of different, new, real-time PCR-based pathogen identification methods although the clinical practicability of such techniques is not yet fully clarified.

The present study focuses on a novel, multiplex, real-time PCR-based pathogen identification system developed for rapid differentiation of the commonly occurring bacterial and fungal causative pathogens of bloodstream infections.

**Results:**

A multiplex, real-time PCR approach is introduced for the detection and differentiation of fungi, Gram-positive (G+) and Gram-negative (G-) bacteria. The Gram classification is performed with the specific fluorescence resonance energy transfer (FRET) probes recommended for LightCycler capillary real-time PCR. The novelty of our system is the use of a non-specific SYBR Green dye instead of labelled anchor probes or primers, to excite the acceptor dyes on the FRET probes. In conjunction with this, the use of an intercalating dye allows the detection of fungal amplicons.

With the novel pathogen detection system, fungi, G + and G- bacteria in the same reaction tube can be differentiated within an hour after the DNA preparation *via* the melting temperatures of the amplicons and probes in the same tube.

**Conclusions:**

This modified FRET technique is specific and more rapid than the gold-standard culture-based methods. The fact that fungi, G + and G- bacteria were successfully identified in the same tube within an hour after the DNA preparation permits rapid and early evidence-based management of bloodstream infections in clinical practice.

## Background

Bloodstream infections are life-threatening, especially in individuals with serious underlying conditions or an impaired immune system [1]. The number of reported cases of bloodstream infections in the USA, between 1979 and 2002, was 10, 319, 418 and demonstrated an annualized increase of 8.7% [2]. In critically ill patients, the majority of infections are caused by bacteria but fungal infections, although these account for only 4.6% of all infections, have a significant impact on public health. [2]. Mixed fungal/bacterial infections are not uncommon, incidences of combined *Candida* and bacterial bloodstream infections have been reported in as many as 23% of all episodes of candidaemia [3]. Despite its relatively low frequency, fungal blood stream infections can progress to severe sepsis and septic shock, associated with a drastic rise in mortality; therefore, early and appropriate treatment of such infections is critical [4,5].

Since molecular diagnosis in sepsis is reliable, and faster than the classical blood-culturing techniques, there has been an increase in interest in methods such as PCR, ligase chain reaction, nucleic acid sequence based amplification, and nested PCR [6,7]. Nevertheless, these molecular approaches are applied only following the positivity of the blood culture; therefore, they require a substantial amount of elapsed time.

In contrast, the LightCycler PCR assay is fast, reliable and relatively easy to perform - even in small laboratories.

This method is based on a previously-reported fluorescence resonance energy transfer (FRET) technique which involves a distance-dependent interaction between the electronic excited states of two dye molecules [8]. The excitation is transferred from a donor (anchor) molecule to an acceptor (quencher) molecule, without emission of a photon, and has been proved to be an appropriate method for discriminating between the commonly occurring pathogen G + and G- bacteria [9]. The differentiation, *via* the melting temperature of the overall PCR product and the melting point of the probes, allowed creation subgroups within the G + and G- stains, and this system required less than 4 h, inclusive of the time need for the DNA preparation and the evaluation of the PCR results [10]. Until now, parallel detection of fungal and bacterial infections in a real-time system has been an unresolved problem however there are several tests in the market with the same purpose. Some of them detect bacteria, without fungal identification (Prove-It; Mobidiag, Helsinki, Finland or SeptiTest; Molzym, Bremen, Germany). The Reflex PCR assay (Molzym, Bremen, Germany) includes several steps after the PCR which increases the time required. The SepiFast (Roche; Basel, Switzerland) assay is similar to our system but works with three parallel reaction vessels and a different principle for detection. Furthermore, it requires individual molecular laboratory, equipments and software. Identification of the most common clinically relevant fungi is possible through a simple melting-point analysis relating to the ITS2 (internal transcribed spacer) region. This non-coding region is a highly variable rRNA region that is adaptable for the identification of clinically relevant fungi over a broad range [11]. Measurements are made at 540 nm, and require a non-specific intercalating dye [12].

Real-time PCR detection can be performed by using free dyes or labelled sequence-specific probes. One combination of the two techniques uses unlabelled probes for the amplicon detection and T_m_ determination [13]. Another parallel application was the combination of TaqMan chemistry and the very new, aspecific dye, BOXTO, as a multiplex PCR [14].

The novelty of our prototype system lies in the use of non-specific SYBR Green dye as a donor molecule, instead of a labelled primer or other specific anchor probe. With this technique, it is possible to examine pathogenic fungi, G + and G- bacteria in a single tube multiplex PCR reaction.

## Results and discussion

### Discrimination of the fungal, G + and G- bacterial pathogens

DNA samples from all species studied were prepared and amplified successfully with the SYBR Green dye-based method in the LightCycler instrument. Species-specific T_m_-s were obtained by melting-point analysis on three detection channels and all pathogens were identified correctly as fungi or G- or G + bacteria (Table 1). On the F1 channel (540 nm), the melting points of all the amplicons (T_m_ A) were visible, due to the fluorescent signal of the SYBR Green non-specific intercalating dye. On the F2 (640 nm) and F3 (705 nm) channels, the G- and the G + probes (T_m_ P), respectively, gave fluorescence signals. After the discrimination of the G- and G + strains, the fungal pathogens could be screened, because the fungal strains gave no signal on the F2; F3 channels.

**Table 1:** Melting points of bacterial and fungal amplicons and probes

**Microbial strains**	**T**_**m **_**P (°C)**		**T**_**m **_**A (°C)**	
**Gram positive (G+)**	Mean	SD	Mean	SD
*Enterococcus faecalis*	67.94	0.07	84.14	0.36
*Enterococcus faecium*	67.84	0.21	84.59	0.78
*Listeria monocytogenes*	67.80	0.19	86.01	0.36
*Staphyloccus aureus*	64.85	0.21	83.91	0.54
*Staphyloccus epidermidis*	64.50	0.30	83.60	0.36
*Streptococcus pyogenes*	46.54	0.56	84.38	0.78
**Gram negative (G-)**				
*Acinetobacter baumannii*	66.09	0.15	82.90	0.16
*Bacteroides fragilis*	48.65	0.18	84.47	0.84
*Enterobacter aerogenes*	63.95	0.34	83.47	0.48
*Enterobacter cloacae*	64.98	0.09	84.38	0.24
*Escherichia coli*	64.69	0.44	84.74	0.54
*Haemophilus influenzae*	61.99	0.35	84.28	0.30
*Klebsiella pneumoniae*	65.13	0.23	84.57	0.20
*Proteus vulgaris*	64.58	0.18	82.87	0.24
*Pseudomonas aeruginosa*	53.32	0.33	83.00	0.34
*Serratia marcescens*	64.01	0.30	84.17	0.30
*Stenotrophomonas maltophilia*	58.10	0.07	84.42	0.15
**Fungi**				
*Candida albicans*	-	-	87.1	0.33
*Candida dubliniensis*	-	-	85.5	0.50
*Candida quillermondii*	-	-	85.1	0.70
*Candida krusei*	-	-	89.8	0.02
*Candida parapsilosis*	-	-	85.4	0.88
*Candida tropicalis*	-	-	84.5	0.75
*Aspergillus fumigatus*	-	-	91.0	0.38

All the amplicons T_m_ were measured at the F1 channel (540 nm). The signal was generated by aspecific SybrGreen dye. The G + specific probes produced a signal at the F2 channel (640 nm) the G- probes at the F3 channel (705 nm). The signals were induced with the help of a special FRET technique.

### Determination of the bacterial pathogens

**Figure 1:**
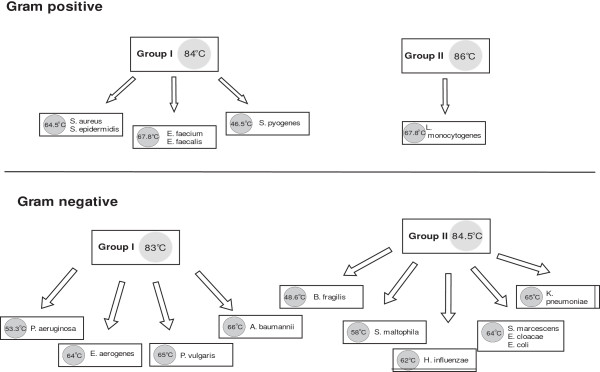
**Differentiation of the bacterial pathogens**. The group temperatures indicate the entire T_m_ of the pathogens. The subgroup temperatures are the melting temperatures of the hybridization probes.

Four G + and nine G- bacterial subgroups could be distinguished through a joint consideration of the melting points of the probes and the melting point of the overall PCR product (Figure 1).

**Figure 2:**
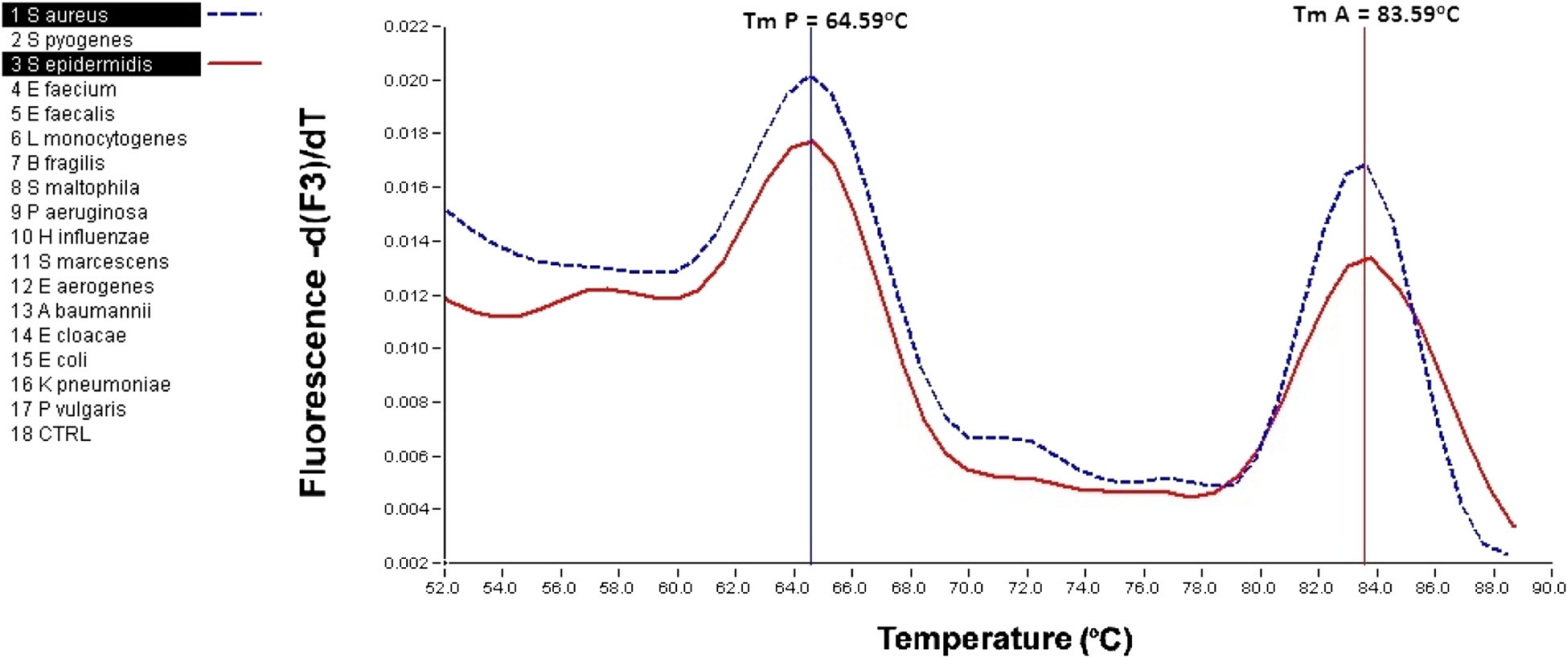
**Melting-peaks of *Staphylococcus aureus *and *Staphylococcus epidermidis.*** Revealing that it is impossible to differentiate these *Staphylococcus* species *via* the T_m_ data of the amplicons or probes.

*S. aureus* and *S. epidermidis* have very close-lying melting temperatures and their species-specific differentiation is not possible *via* this 16S rRNA sequence (Figure 2). A comparison of the Gene Bank sequences (*S. aureus* and *S. epidermidis* NCBI Taxonomy ID: NC_009782.1 and JF_799903.1) of these species revealed a variance of only three base-pairs, none of them in the region where the probe is associated with the DNA. Thus, determination of the clinically relevant *Staphylococcus* species requires other gene sequences in which the antibiotic resistance can be detected [15]. The situation is the same for the two *Enterococcus* species [16]. At the same time, *S. pyogenes* and *L. monocytogenes* are clearly differentiable.

**Figure 3:**
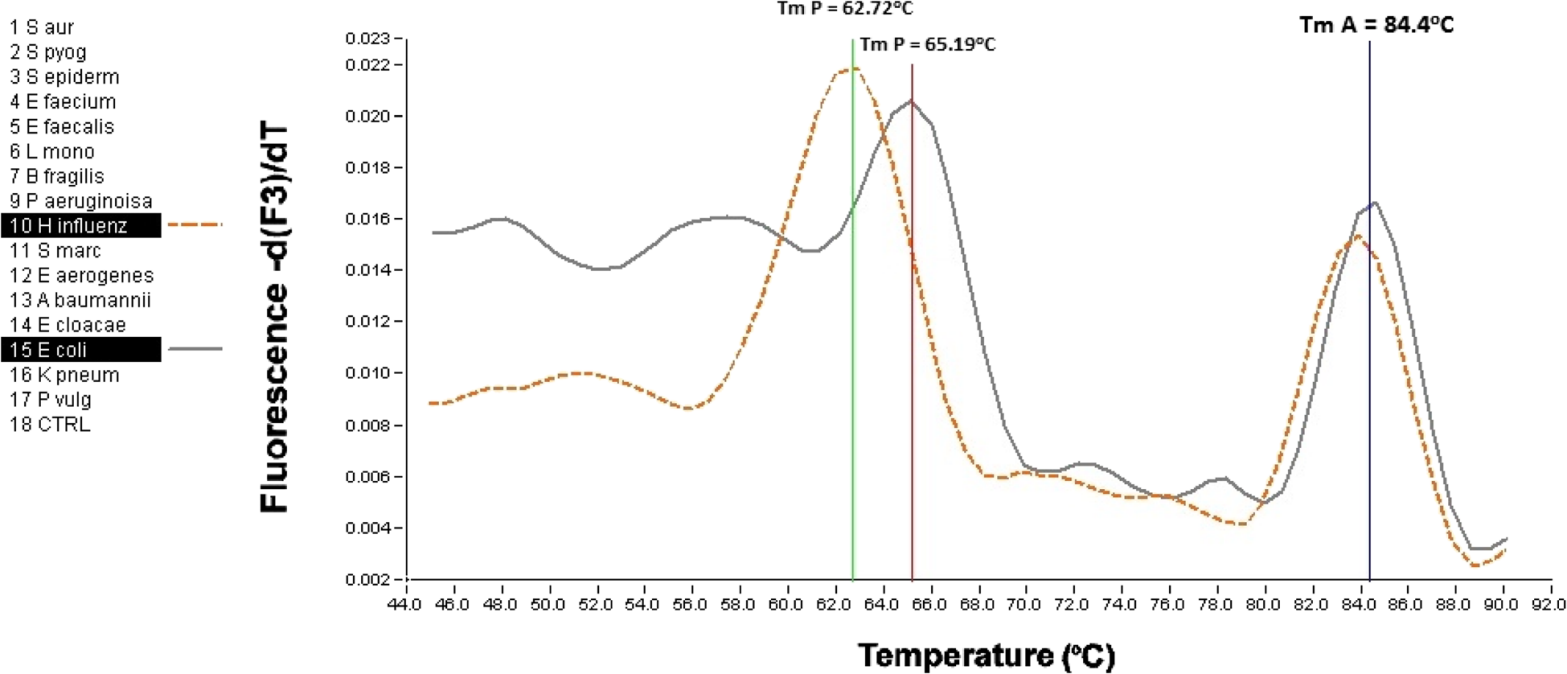
**Differentiation of *Escherichia coli *from *Haemophilus influenzae*.** Although these pathogens have a very similar T_m_ in the 16S rRNA region, the T_m_ of the probes are clearly different.

Among the G- bacteria, *E. coli* is one of the most common causative agents of bloodstream infections [17]. Unfortunately, it has almost the same T_m_ as those of *E. cloacae* and *S. marcescens*. Other bacterial strains, such as *H. influenza*, are clearly differentiable through the melting temperature of the probe (Figure 3) or amplicon. The sensitivity of the reaction was five colony-forming units (CFU) per reaction.

### Determination of fungal pathogens

Fourteen frequently-encountered fungal pathogens could be distinguished. The highly variable ITS 2 target sequence allowed correct identification of all of the clinically relevant fungal strains, through the T_m_ points on the F1 channel [12,18]. There was no signal on the F2 or F3 channel. The sensitivity of the reaction was 5 CFU per reaction.

The correct differentiation between bacteria and fungi was verified by means of gel electrophoresis, with the help of the amplicon length (fungal amplicons 192–494 bp, bacterial 187 bp).

### Determination of the co-infection model

In case of co-infections, there are some limitations in the detection. If the ratios of the different agents are higher than 1:10, the system does not detect the infectious agent which is in lower quantities.

### Calibration of the multiplex PCR

All three non-specific dyes (LCGreen, EvaGreen and SybrGreen) excited all of the labelled probes (LCRed640, LCRed705 and Cy5.5). The best results were obtained with the SybrGreen dye.

The determination of T_m_ is very sensitive to the composition of the PCR reaction mixture, and primarily to the ionic strength. To avoid T_m_ bias originating from pipetting errors between PCR runs, the application of mastermixes is advisable. One limitation of the method is that the various mastermixes offered by different suppliers differ in reagent composition, which may influence the T_m_ values.

Naturally, repeated runs with a given mastermix yield reproducible data. In the event of a change of mastermix, however, calibration is necessary to establish the new T_m_ data on the fungal strains.

The data determined in the present work were obtained with the use of “Fermentas Maxima SybrGreen, no ROX” and five-eight parallel experiments. No false positive samples were found when this method was tested. No significant differences in the melting peak temperatures were observed between different isolates of the same species. The standard deviation of the melting peak temperatures of all 21 references and 93 clinical isolates with bacterial and fungal strains was between 0.08 and 0.88, as listed in Table 1. These data are in concordance with our previous results [19,20].

### Sensitivity and reproducibility

For sensitivity testing of the prototype system, six bacterial and two fungal gDNA preparations were made from artificially infected blood. Eight species, and eight parallel investigations of five dilutions of the bacterial suspensions in blood were, analysed. Of 8 reactions for each species, all were positive with 50 DNA copies, 98.5% were positive with 10 copies, 67.2% were positive with 5 copies and 21.9% were positive with 2 copies (Table 2). All the reactions were carried out within the same parameters described in the section PCR conditions.

**Table 2:** Diagnostic sensitivity of the PCR

**Microbial strains**	No. (%) of positive PCRs*				
**Gram positive (G+)**	50 copies	10 copies	5 copies	2 copies	1 copy
*Enterococcus faecalis*	8 (100)	8 (100)	5 (62.5)	2 (25)	0 (0)
*Staphylococcus aureus*	8 (100)	8 (100)	7 (87.5)	3 (37.5)	0 (0)
*Streptococcus pyogenes*	8 (100)	8 (100)	5 (62.5)	5 (62.5)	0 (0)
**Gram negative (G-)**					
*Enterobacter aerogenes*	8 (100)	8 (100)	5 (62.5)	2 (25)	0 (0)
*Escherichia coli*	8 (100)	8 (100)	6 (75)	1 (12.5)	0 (0)
*Haemphilus influenzae*	8 (100)	7 (87.5)	4 (50)	0 (0)	0 (0)
**Fungi**					
*Candida albicans*	8 (100)	8 (100)	5 (62.5)	0 (0)	0 (0)
*Candida tropicalis*	8 (100)	8 (100)	6 (75)	1 (12.5)	0 (0)

*Out of 8 samples.

Three Gram positive, three Gram negative and two fungal strains were used for the infection of healthy donor bloods. All the experiments were carried out eight times using 5 dilutions of the pathogens.

## Conclusions

Real-time PCR is one of the fastest diagnostic methods currently available. The use of rRNA genes for the detection is based on the conserved 16S rRNA sequences of the bacteria. As regards fungi, the ITS sequence refers to a segment of non-functional RNA, situated between 5.8S and 28S rRNAs. To reproduce the results, it is possible to differentiate between fungi and bacteria, or between fungal species by electrophoresis [21,22] or melting-point analysis [23]. The Roche LightCycler PCR was specially developed to amplify amplicons under 500 bp. The amplicons amplified by PLK1/PLK2 comprised 187 bp, while the fungal amplicons amplified by ITS86/ITS4 primer pair varied between 192 bp (*Geotrichum candidum*) and 494 bp (*Malassezia furfur*), values which are perfectly suited to this instrument profile. In this study, the advantage of the LC system was utilised when FRET technique was used to detect and differentiate the bacterial pathogens. As a novel element, excitation of the fluorescent probes was carried out with the help of a non-specific intercalating dye, this is an uncommon procedure in real-time investigations. It allows parallel detection of fungal pathogens and with bacteria in the same tube. As the result of the use of the multiplex PCR in combination with FRET probes and melting point-analysis, the broad-range identification of many frequent causative agents of bloodstream infections becomes possible within four hours. Sensitivity of pathogen PCR in sepsis is generally between 3 and 100 CFU/mL according to the literature [24]. The sensitivity of our prototype system was five CFU per reaction, which in combination with an efficient preparation is suitable for the detection of bloodstream infections. If commercially available “Midi” preparation kits (i.e.: NucleoSpin Blood L, Macherey-Nagel, Düren, Germany) were used, the sample mateial was 2 mL of blood, the elution volume was 100 μL and finally 5 μL of eluate were used for subsequent PCR. The calculated sensitivity was 50 CFU/mL blood. The sample/eluent ratio was the same in case of midi and maxi preparation kits which means that increased sample volume is not enhancing the sensitivity [25]. The sensitivity of the “gold standard” conventional blood culture technique is one CFU per 10 mL blood sample. Our method is less sensitive. The blood culture technique is not replaceable with molecular techniques so far but the time delay until the adequate therapy can be reduce.

To determine the diagnostic sensitivity and reproducibility of the method, experiments with artificially infected blood were performed. The sensitivity of the PCR was 2 to 10 copies per reaction, which was the same as with cultivated cells. The melting points (TmA and TmP) were the same as we described in Table 1. with “Fermentas Maxima SybrGreen, no ROX”; therefore, human gDNA does not inhibit the reaction and does not modify the melting peaks.

With this method, neither the G + *S. aureus* and *S. epidermidis* nor the G- *E. coli*, *E. cloacae* and *S. marcescens* can be distinguished, and additional species-specific probes or primers are necessary for the further differentiation of these species.

Antibiotic resistance cannot be determined directly with this prototype system. The susceptibility testing of resistant *E. coli* strains can be performed using a PCR-based technique with other 16S rRNA specific primers [26]. Unfortunately, these investigations require a PCR analysis after the identification of the bacteria.

In spite of its limitations, the prompt and reliable information provided by this new diagnostic method on the most common pathogenic bacteria might permit targeted therapy with narrow-spectrum antibiotics, instead of empirically-administered broad-spectrum antibiotics. To confirm these findings in clinical practice, a prospective study is now being designed and engineered.

The incidence of sepsis has been continuously increasing over recent decades, and the early detection of the pathogens can have a great impact on the clinical outcome of infections [27-30] Molecular diagnostic systems allow species identification in less than 24 hours - which is a drastic improvement relative to the gold-standard, culture-based method and Gram staining-based identification methods that yield results in 24 to 72 hours [31,32].

With the novel method described above (multiplex PCR with the new combination of aspecific dyes and labelled probes), the most common causative agents of bloodstream infections can be detected in two hours, without DNA preparation; therefore, this method offers a huge advantage over traditional FRET-based assays by accurately detecting the T_m_ of both the probes and the amplicons.

## Methods

Reference strains of 17 clinically relevant bacterial species were collected, as typical of the main causative agents of bloodstream infections [33]. Nine reference strains, *Staphylococcus aureus* ATCC 25923*, Staphylococcus epidermidis* ATCC 12228*, Enterococcus faecalis* ATCC29212*, Listeria monocytogenes* ATCC 4701, *Bacteroides fragilis* ATCC 25285*, Pseudomonas aeruginosa* ATCC 27853*, Haemophilus influenzae* ATCC 49247*, Escherichia coli* ATCC 25922 and *Klebsiella pneumoniae* ATCC 700603 were from the American Type Culture Collection. [ATCC]*. Streptococcus pyogenes* OKI 80002 was from the National Centre for Epidemiology, Hungary [OKI] and *Proteus vulgaris* HNCMB 60076 was from the Hungarian National Collection of Medical Bacteria [HNCMB]. Furthermore, to confirm the reliability and reproducibility of the technique, clinical strains of *S. aureus* (n = 4), *S. epidermidis* (n = 6), *S. pyogenes* (n = 2), *E. faecalis* (n = 2), *E. faecium* (n = 3)*, L. monocytogenes* (n = 1), *B. fragilis* (n = 2), *P. aeruginosa* (n = 1), *H. influenzae* (n = 1), *E. coli* (n = 5), *K. pneumoniae* (n = 5), *P. vulgaris* (n = 3), *Stenotrophomonas maltophilia* (n = 2)*, Serratia marcescens* (n = 2)*, Enterobacter aerogenes* (n = 2)*, E. cloacae* (n = 2) and *Acinetobacter baumannii* (n = 3) from the Institute of Clinical Microbiology at the University of Szeged were also included. The species identities of the clinical isolates were confirmed by conventional biochemical methods.

Ten fungal strains were examined. Five reference strains, *Candida albicans* ATCC 10231 and ATCC 14053, *C. tropicalis* ATCC 750, *C. parapsilosis* ATCC 22019 and *C. glabrata* ATCC 39316, were from the [ATCC], *Cryptococcus neoformans* IFM 5844 and IFM 5855 were from IFM Quality Services Pty Ltd [IFM], and *Aspergillus fumigatus* SzMC 2486, *A. flavus* SzMC 2536 and *A. niger* SzMC 2761 were from the Szeged Microbiological Collection [SzMC]. Furthermore, clinical strains of *C. albicans* (n = 14), *C. glabrata* (n = 5), *C. tropicalis* (n = 4), *C. parapsilosis* (n = 5), *C. krusei* (n = 4), *C. quillermondii* (n = 4), *C. lusitaniae* (n = 3), *C. norvegensis* (n = 1), *C. inconspicua* (n = 2), *C. dubliniensis* (n = 2) and *Cryptococcus neoformans* (n = 2) from the Institute of Clinical Microbiology at the University of Szeged were also tested.

### Bacterial DNA purification

The bacterial strains were grown on Columbia agar base under aerobic conditions, except that *Bacteroides fragilis* was grown under anaerobic conditions. The bacterial DNA was extracted with the QIAamp® DNA Blood Mini Kit (QuiaGene Inc, Chatsworth, Calif., USA), following the manufacturer’s instructions in “Protocols for Bacteria”. One millilitre of log-phase culture suspension, at a concentration of 10^7^ CFU/mL, was used for the preparation. For determination of the sensitivity of the reaction, 100 μL of the serially diluted *S. aureus* reference strain was used for DNA extraction. The number of bacterial cells was determined by plating aliquots of serially diluted samples onto Columbia agar base.

For lysis of the rigid multilayered G + bacterial cell wall, we used a pre-incubation step with 20 mg/mL lysozyme (in 20 mM Tris · HCl, pH 8.0, 2 mM EDTA, 1.2% TritonX100). The spin protocol for “DNA Purification from Tissues” was followed, after incubation at 30°C for 30 min. The final concentration of DNA was 2.0-13.8 ng/μL, with a ratio A260/A280 = 1.6-1.8 after purification.

### Fungal DNA purification

All the fungi were grown on Sabouraud medium. The fungal DNA was extracted from 1 mL of a log-phase culture suspension containing 9.6 × 10^7^ of fungal cells. For determination of the sensitivity of the reaction, 100 μL of the serially diluted *C. albicans* reference strain was used for DNA extraction. The number of fungal cells was determined by plating aliquots of serially diluted samples onto Sabouraud-glucose medium.

We followed the QIAamp® DNA Mini Kit Protocol for Yeasts. In this case, additional reagents were required for elimination of the complex fungal cell-wall structure: sorbitol buffer (1 M sorbitol, 100 mM EDTA, 14 mM β-mercaptoethanol) [34] was used, and the samples were incubated with lyticase for 30 min at 30°C. Efficient and complete lysis was achieved in 1.5 hour in a shaking water-bath. This purification yielded 2.0–25 μg of DNA in 100 μL of water (2.0–13.8 ng/μL), with A260/A280 = 1.6–1.8.

### DNA preparation from infected blood

Samples of 180 μL healthy donor bloods in EDTA vacutainer tubes were infected with 20 μL of log-phase culture suspension at a concentration of 10^8^ CFU/mL bacterial and/or fungal suspensions. Bacterial and fungal cells were quantified, in a Bürker chamber, by viable counts. For the sensitivity testing of the prototype system, the bloods were infected with five dilutions of the log-phase culture suspension at a final volume of 20 μL. The first dilution contained 50 copies in 1 μL template DNA (2.5x10^4^ CFU/mL blood), the second contained 10 copies (5x10^3^ CFU/mL blood), the third 5 copies (2.5x10^2^ CFU/mL blood) and the fourth 2 copies (5x10^2^ CFU/mL blood). The red blood cells were disrupted by lysis buffer [35], the bacterial and fungal cell wall lysed using the freezing-thawing method. After digestion with Proteinase K, the DNA was extraction carried out as reported previously [36].

### Bacterial and fungal primer design, FRET probes

Two primer pairs were used for multiplex amplification of bacterial and fungal DNA.

The bacterial primer pair was PLK1 (TAC GGG AGG CAG CAG) forward and PLK2 (TAT TAC CGC GGC TGC T) reverse, which are highly conserved in different groups of bacteria [9] and amplify the 16S rRNA sequence. The PLK2 reverse primer was modified and used without the inner fluorescence labelling. Originally, the labelled primer excited the Gram specific probes. We applied the non-specific SYBR Green dye for excitation; it also serves for visualization of the fungal amplicons. This primer-pair produces a 187 bp fragment in each species.

Previously hybridization probes were used for the Gram classification [10]. ISN2 (5′-CCG CAG AAT AAG CAC CGG CTA ACT CCG T-3′) labelled with LCRed 640 was specific for G-, and ISP3 (5′-CCT AAC CAG AAA GCC ACG GCT AAC TAC GTG-3′) labelled with Cy5.5 was specific for G + bacteria, and the [10] ISP2 probe was labelled with LCRed705 at the 5′ end. The producers offered Cy5.5 dye instead of LCred705. This modified probe was used in our experiments.

The ITS86 forward (GTG AAT CAT CGA ATC TTT GAA C) and the ITS 4 reverse (TCC TCC GCT TAT TGA TAG C) primers were used for detection of the fungi. These primers amplify a 192–494 bp sequence of ITS2 region, which is a highly variable part between the 5.8S and 28S rRNA sequence [37].

### Mastermixes/excitation dyes

Different, non-specific intercalating dyes are used for real-time PCR investigations. Most of these are accessible in ready-to-use, mastermix formulae. Our goal was to choose the best dye for excitation of the labelled probes. The tested dyes were LCGreen “LightCycler® 480 High Resolution Melting Master” (Roche Diagnostic GmbH, Mannheim, Germany); SybrGreen “LightCycler® 480 DNA Master SYBR Green I”, (Roche); “IQ™ SYBR® Green Supermix” (Bio-Rad Laboratries, Inc., Hercules, CA, USA); “Maxima™ SYBR Green qPCR Master Mix no ROX” (Fermentas, Vilnius, Lithuania); and EvaGreen (“LC-FastStart DNA Master Hybridization Probes” (Roche) combined with EvaGreen dye (Biotium Inc., Hayward, CA, USA) and “Sso Fast™ EvaGreen® Supermix” (BioRad). All mastermixes were used according to the manufacturer’s instructions.

### PCR conditions

PCR was performed using a LightCycler real-time PCR instrument (Roche). The reaction volume of 10 μL contained 1 μL of DNA (with a final concentration of ~10 ng/μL), 1 μM of each of the primers, 0.7 μM of each of the probes, an appropriate amount of mastermix, and 0.2 mM BSA (in the cases of the Fermentas and BioRad mastermixes).

The PCR conditions were as follows: initial denaturation at 95°C for 600 s, followed by 40 cycles of denaturation (95°C for 0 s, 20°C/s), annealing (55°C for 15 s, 20°C/s), and extension (72°C for 20 s, 2°C/s). The emitted fluorescence was measured after the annealing steps. The melting-curve analysis procedure consisted of 1 cycle at 95°C for 10 s, 40°C for 120 s, followed by an increase in the temperature to 95°C at 0.2°C/s. The fluorescence signal (F) was monitored continuously during the temperature ramp, and plotted against temperature (T).

### Data analysis

The melting peaks were evaluated using the LightCycler Software V 3.5. The melting-peaks were determined through the manual T_m_ option on the three detection channels (F1, F2 and F3).

The standard deviation (SD) of the melting-points was calculated from five parallel experiments. The fungal or bacterial samples were verified by gel electrophoresis on 1.5% agarose gel, with the help of a low-range DNA ladder.

The sensitivity of the multiplex PCR calculated from five dilutions of the bacterial suspension.

## Abbreviations

RT PCR: Real-time PCR; FRET: Fluorescence resonance energy transfer; LC: LightCycler; ICU: Intensive Care Unit; ITS: Internal transcribed spacer; ATCC: American Type Cell Culture; IFM: IFM Quality Services Pty Ltd; SzMC: Szeged Microbiological Collection; Tm: Melting temperature; SD: Standard deviation; Tm A: Melting peak of the amplicon; Tm P: Melting peak of the probe.

## Competing interests

The authors declare that they have no competing interests.

## Authors’ contributions

ÁH: helped in the design, performed the experiments, analysed the data and wrote the manuscript. ZP: provided the clinical samples, helped in the analysis and interpretation of the data and revised the manuscript. EU: provided all the clinical bacterial samples and critiqued the manuscript. CsV: have made substantial contributions to concept and design, provided the fungal samples and revised the manuscript. FS: designed all the experiments, participated in the writing of the manuscript, revised the manuscript and gave final approval of the version to be published. All the authors have read and approved the final manuscript.

## Authors’ information

ÁH: Doctoral fellow in the Department of Medical Microbiology and Immunobiology, Faculty of Medicine, University of Szeged, Dóm tér 10, Szeged, Hungary, ZP: Chief of Medicine on the Department of Anaesthesiology and Intensive Therapy, Faculty of Medicine, University of Szeged, Semmelweis u. 6, Szeged, Hungary, EU: Chair of the Institute of Clinical Microbiology, Faculty of Medicine, University of Szeged, Semmelweis u. 6, Szeged, Hungary CsV: Chair of the Department of Microbiology, Faculty of Science and Informatics, University of Szeged, Közép fasor 52, Szeged, Hungary FS:Senior research fellow on the Department of Medical Microbiology and Immunobiology, Faculty of Medicine, University of Szeged, Dóm tér 10, Szeged, Hungary,
